# Visual task enhances spatial selectivity in the human auditory cortex

**DOI:** 10.3389/fnins.2013.00044

**Published:** 2013-03-27

**Authors:** Nelli H. Salminen, Joanna Aho, Mikko Sams

**Affiliations:** ^1^Brain and Mind Laboratory, Department of Biomedical Engineering and Computational Science, Aalto University School of ScienceEspoo, Finland; ^2^Brain Research Unit, O.V. Lounasmaa Laboratory, Aalto University School of ScienceEspoo, Finland

**Keywords:** sound source localization, spatial hearing, attention, magnetoencephalography, stimulus-specific adaptation, auditory cortex

## Abstract

The auditory cortex represents spatial locations differently from other sensory modalities. While visual and tactile cortices utilize topographical space maps, for audition no such cortical map has been found. Instead, auditory cortical neurons have wide spatial receptive fields and together they form a population rate code of sound source location. Recent studies have shown that this code is modulated by task conditions so that during auditory tasks it provides better selectivity to sound source location than during idle listening. The goal of this study was to establish whether the neural representation of auditory space can also be influenced by task conditions involving other sensory modalities than hearing. Therefore, we conducted magnetoencephalography (MEG) recordings in which auditory spatial selectivity of the human cortex was probed with an adaptation paradigm while subjects performed a visual task. Engaging in the task led to an increase in neural selectivity to sound source location compared to when no task was performed. This suggests that an enhancement in the population rate code of auditory space took place during task performance. This enhancement in auditory spatial selectivity was independent of the direction of visual orientation. Together with previous studies, these findings suggest that performing any demanding task, even one in which sounds and their source locations are irrelevant, can lead to enhancements in the neural representation of auditory space. Such mechanisms may have great survival value as sounds are capable of producing location information on potentially relevant events in all directions and over long distances.

## Introduction

The way the auditory system represents location constitutes a major deviation from how space is represented in other sensory modalities. In the cortex, neurons form topographical maps of visual and somatosensory locations but for audition, no such map has been shown to exist (see, for instance, Werner-Reiss and Groh, 2008). Also unlike in vision and touch, the input the auditory system receives is not inherently spatial in nature: the perception of sound source location is based on subtle acoustical cues that the path from the source to the ears imposes on the sound (Middlebrooks and Green, 1991). A topographical map of auditory space could still be generated through neural computations but this does not appear to take place (Grothe et al., 2010). Instead, the auditory cortex uses a population rate code for representing horizontal sound source location (Stecker et al., 2005; Werner-Reiss and Groh, 2008; Salminen et al., 2009, 2012; Magezi and Krumbholz, 2010; Briley et al., 2013). In this hemifield or opponent code, horizontal sound source location is represented in the activity of two widely tuned populations, one preferring sounds in the left and the other those in the right hemifield of the auditory space.

The population code of sound source location is influenced by the state of the subject. In a recent study on the auditory cortex of behaving cats, the spatial selectivity of single neurons depended on task conditions (Lee and Middlebrooks, 2011). When the cat was engaged in a listening task, the spatial receptive fields were smaller than during idle listening. More specifically, neurons that had very wide receptive fields, sometimes spanning nearly the entire auditory space, became narrower resulting in receptive fields the size of a little less than half the auditory space. This means that the neurons had a higher level of spatial selectivity during the task than during idle listening. This increase in the number of neurons producing useful information on spatial location occurred both during pitch and location detection tasks although the increase was slightly larger when the task required location detection. The results of a previous study combining magnetoencephalography (MEG) and functional magnetic resonance imaging (fMRI) techniques suggests that such changes in auditory spatial sensitivity may also take place in the human auditory cortex (Ahveninen et al., 2006). Responses were recorded to changes in sound source location and the amplitude of the response was used as a measure of neural selectivity to location. The subjects performed tasks that required the detection of sound source location or the identity of a speech sound. During the spatial task, response amplitudes to changes in sound source location were larger than during the speech task. This suggests that neural selectivity to spatial location was at a higher level when the subjects paid attention to sound source location.

Another form in which neural responses to spatial sound may change due to task demands has been described in previous electroencephalography (EEG) studies. When sounds occur in locations to which attention is directed, they lead to larger response amplitudes than sounds that occur in unattended locations (Hillyard et al., 1973; Teder-Sälejärvi and Hillyard, 1998). A similar gain effect can also be induced crossmodally. For instance, when attention is directed to visual stimuli in a given location, sounds occurring in the same location induce larger negativity in the event-related potentials (ERPs) than sounds occurring outside the focus of visual attention (Eimer and Schröger, 1998; Teder-Sälejärvi et al., 1999). It is not clear whether this increase in response amplitude is due to the activity of spatially selective neurons and whether the widely-tuned neurons typically found in the auditory cortex are even capable of producing increases in response amplitude specifically for a single sound source direction. Since no neurons in the auditory cortex appear to be dedicated to narrow ranges of locations, such increments could not be generated by simply increasing the activity level in a subset of spatially selective neurons.

In summary, the state of the subject can induce two kinds of changes in cortical activity to spatial sound. First, performing a task related to sounds and their source locations enhances spatial selectivity. This enhancement does not appear to occur exclusively for a single attended location but instead applies to all spatial locations equally, although this has not been tested explicitly in previous studies. Second, focused spatial attention leads to a gain effect, that is, an increase in response amplitudes specifically for sounds occurring in an attended spatial location. The gain effect can be induced also crossmodally. However, the enhancement in spatial selectivity has been demonstrated only during auditory tasks. Therefore, the goal of the present study was to establish whether the enhancement in selectivity to sound source location in auditory cortex can be induced crossmodally by a visual task. Additionally, we explored the possibility that this enhancement may depend on the direction of visual orientation. To this end, we evaluated spatial selectivity in the human auditory cortex utilizing an adaptation paradigm in MEG. The same auditory stimulation was repeated during visual tasks and while no task was performed.

## Materials and methods

### Subjects

Fourteen subjects (*mean* age = 24, *SD* = 2.8, 7 female) with normal hearing and normal or corrected-to-normal vision took part in the experiment. Written informed consent was obtained from each participant and the experiments were approved by the Ethical Committee of Helsinki University Central Hospital. The data of one subject were discarded due to poor signal-to-noise ratio.

### Stimuli and procedure

The sound stimuli were 200 ms bursts of white noise presented through headphones. Three virtual locations in the horizontal plane, one to the left (−20°), one directly in front (0°), and one to the right (+20°) were generated by filtering the sounds with corresponding head-related transfer functions (from a database provided by Massachusetts Institute of Technology: http://sound.media.mit.edu/resources/KEMAR.html).

**Figure 1:**
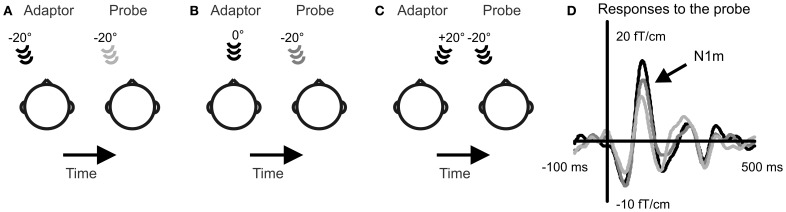
**Selectivity to sound source location in the human cortex was recorded with a stimulus-specific adaptation paradigm.** A probe sound was presented to the left of the subject and adaptor sounds to the left **(A)**, in front **(B)**, and to the right **(C)**. Event-related fields were recorded to the probe sound. The N1m response amplitude increased with growing spatial separation between the probe and the adaptor **(D)**. Event-related fields averaged over 13 subjects and over the three task conditions are depicted from the gradiometer showing largest N1m amplitudes over the right auditory cortex.

The sound stimulation followed a stimulus-specific adaptation paradigm (Butler, 1972; Salminen et al., 2009). In this paradigm, sounds are presented from two alternating locations: the probe and the adaptor, and the effect the location of the adaptor has on the response to the probe sound is recorded (Figure 1). The probe remains in the same location while the adaptor location is varied. When the two sounds are presented in the same location, small response amplitudes are found for the probe sound. However, when a spatial separation is introduced between the sound source locations, the probe gives rise to larger response amplitudes. This can be interpreted in terms of spatial selectivity of auditory cortical neurons. When the probe and the adaptor are presented in the same location they activate the same population of spatially selective neurons. This leads to high levels of attenuation and small responses. When the probe and the adaptor occur in different locations, they activate a partially different set of spatially selective neurons. Then, the spatially selective neurons are more likely to respond differently to the two sounds and experience a longer interstimulus interval. This leads to less attenuation and larger response amplitudes. In this study, the probe was always at −20° while the location of the adaptor was either −20°, 0°, or +20°. Each adaptor condition was confined to a dedicated stimulation block resulting in three blocks for each task condition. Sounds were presented at an interstimulus-interval (onset to onset) of 1 s.

During the visual task conditions, subjects were presented with visual stimuli projected on a screen at a distance of 93 cm from the subject. The stimuli were solid-color circles of 3.7° diameter located 20° to the left or right of the midline. The circles were presented sequentially for a duration of 150 ms at an interstimulus interval of 650 ms. The timing of the visual stimuli was arranged so that their onset did not coincide with the onset of the sounds. Six colors were used (white, red, purple, blue, green, and yellow) and the task was to indicate by a button press when two subsequent circles of the same color occurred. Attention was maintained in the same direction (left or right) throughout each stimulation block. The subjects were encouraged to overtly orient to the visual stimuli by directing their gaze either to the left or right. A cross was presented directly in front and the subjects were instructed to orient their head so that their nose was pointing toward the cross. Head position was monitored with coils attached to the head. In an additional control condition, the subjects read a self-selected text and were instructed to ignore all stimulation. The purpose of this condition was to record brain responses while the subject was in a relaxed state but still maintaining sufficient alertness for brain recordings. This provided the possibility of evaluating the contribution of general attentive state of the subject. During all conditions, the subjects were instructed to ignore the sound stimulation.

The experiment comprised altogether nine stimulation blocks: the three adaptor conditions (−20°, 0°, or +20°) were repeated for each task condition (left, right, control). The experiment always began with three task blocks, continued with three control blocks and finally ended with the three remaining task blocks. Within these restrictions, the order of presentation was randomized for each subject.

### Data acquisition and analysis

Data was acquired with a 306-channel MEG device (Vectorview, Elekta Neuromag, Finland) at a sample rate of 1000 Hz and a passband of 0.03–200 Hz. The event-related fields were averaged online from 100 ms prior to stimulus onset to 500 ms after. Large eye-movements and blinks were monitored with electrodes and deviations larger than 150 μV in the electrodes or 3000 fT in the MEG data led to the automatic discarding of the epoch. A minimum of 150 repetitions were obtained for each stimulus condition. The event-related fields were then filtered at 1–30 Hz and baseline corrected with respect to a 100-ms prestimulus period.

To quantify the overall level of activity in the auditory cortex, three pairs of planar gradiometers above the auditory cortex with the largest levels of activity around the N1m latency range were chosen for further analyses for each subject and cortical hemisphere separately. The N1m amplitude was quantified as the amplitude peak at the latency range of 80–140 ms in the average waveform of the three channel pairs. All data analysis was performed on these amplitudes. For illustration purposes, data was also averaged over all 13 subjects and the gradiometer showing largest N1m responses was identified in the right hemisphere.

The N1m amplitudes were submitted to a repeated-measures analysis of variance with the repeating factors task condition (left, right, control) and adaptor location (left, front, right). Newman–Keuls *post-hoc* comparisons were performed when appropriate. The analyses of left-hemispheric activity did not yield any findings that reached or approached statistical significance. This was possibly due to the probe sound being always in the left hemifield resulting in low levels of activity in the ipsilateral left cortical hemisphere. The right auditory cortex has been suggested to be more involved in spatial processing (Zatorre and Penhune, 2001; Palomäki et al., 2005) and this may further account for the lack of systematic variations in the left-hemispheric responses. Therefore, only right-hemispheric activity is discussed in the results.

## Results

The response amplitude to the probe sound depended on the location of the adaptor indicating neural selectivity to sound source location [Figure 1; main effect of adaptor location: *F*_(2, 24)_ = 20.7, *p* < 0.001]. Smallest responses occurred when the adaptor was at the same location with the probe in the left (−20°) and largest when the adaptor was in the right hemifield (+20°). The response amplitude was intermediate for the adaptor location directly in front. The increase in response amplitude with growing spatial separation occurred both during the visual task and when no task was performed. This pattern of location-specific adaptation is in line with previous experiments evaluating spatial selectivity with the adaptation paradigm (Butler, 1972; Salminen et al., 2009).

**Figure 2:**
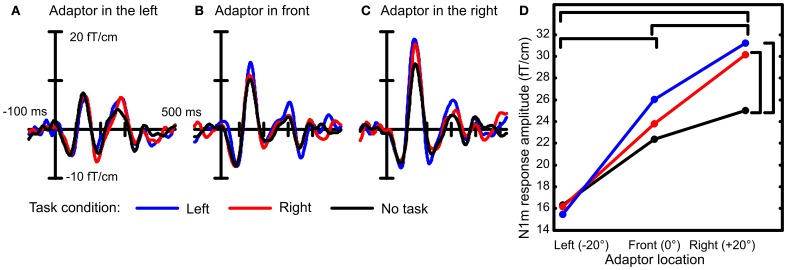
**Spatial selectivity was recorded under three conditions: when a visual task required orientation to the left or to the right and while the subject ignored all stimulation.** Example event-related fields are depicted from the right-hemispheric gradiometer showing largest N1m amplitudes averaged over 13 subjects. When the probe and the adaptor were both presented in the left location, the three task conditions led to similar amplitudes of the N1m response **(A)**. When there was a spatial separation between the probe and the adaptor, larger responses to the probe were recorded during the visual task conditions than when no task was performed (**B** and **C**). Therefore, the increase in N1m response amplitude to the probe was larger during the visual task condition than when the subject was not engaged in a task **(D)**. This suggests that the spatial selectivity of auditory cortical neurons was enhanced during the performance of the visual task. Statistically significant differences are marked with brackets.

The task condition influenced the rate at which the response amplitudes increased as a function of spatial separation between the probe and the adaptor [Figure 2; interaction between adaptor location and task condition: *F*_(4, 48)_ = 2.8, *p* < 0.05]. The largest difference between the task conditions was found when the adaptor was in the right hemifield (at +20°), i.e., at the location furthest from the probe. Then, the response to the probe was 33% larger during the performance of the visual task than when all stimulation was ignored (no task vs. right: *p* < 0.01, no task vs. left: *p* < 0.01, right vs. left: *p* = n.s.). A similar but smaller effect (10–20%) was found for the intermediate adaptor location in front but this effect did not reach statistical significance. There was a small difference between the two visual task conditions but this also failed to reach significance (*p* = 0.37). When the adaptor was at the same location as the probe, no differences between task conditions occurred. In conclusion, spatial selectivity, signaled here by location-specific adaptation, was enhanced during the performance of the visual task.

Interestingly, when both sounds were in the same location in the left, the response amplitudes were at the same level under all task conditions. This has two implications for the interpretation of the results. First, the response amplitude to the sound in the left did not depend on whether visual orientation was to the left or to the right. This signifies that no gain effect occurred for the attended location. Second, there was no general increase in response amplitudes that would apply to all sounds during the performance of the task compared to idle listening. Therefore, the changes in response amplitudes occurring during task performance signaled enhancement in spatial selectivity rather than a general increment in neural responsiveness.

## Discussion

Here, we conducted an MEG experiment in order to determine whether orienting to visual stimuli could enhance the selectivity of auditory cortical neurons to sound source location. We measured responses to spatial sounds in a stimulus-specific adaptation paradigm while subjects were engaged in a visual task. As in previous studies (Butler, 1972; Salminen et al., 2009), the growing spatial separation between sound sources led to an increase in response amplitudes reflecting neural selectivity to spatial location. This indicator of spatial selectivity was modulated so that the rate at which the response amplitudes increased as a function of spatial separation was higher during the visual task than when no task was performed. This suggests that attending to visual stimulation can enhance the spatial selectivity of auditory cortical neurons. This also provides an interesting demonstration on how attention in one modality can modulate the neural coding of stimulus features in another modality.

The enhancement in spatial selectivity found here could possibly be explained by single neuron mechanisms similar to those described in a previous study of cat auditory cortical activity. In the previous study, the spatial receptive fields were found to change in response to engaging in a listening task compared to idle listening (Lee and Middlebrooks, 2011). During idle listening, the receptive fields were wide, often covering more than half the auditory space. However, when the cat performed an auditory detection task, either related to sound source location or pitch, some spatial receptive fields became smaller, typically the width of a little less than half the auditory space. Such a change in the spatial receptive fields, if it took place also during a visual task, could account for the present findings. The narrowing of the spatial receptive fields would lead to a situation in which neurons that during idle listening are hardly selective to spatial location become spatially selective during the performance of a task. Then, more neurons would show spatial selectivity that contributes to the stimulus-specific adaptation to spatial location. This would give rise to a larger adaptation effect during active than passive conditions as was found here. Therefore, the present findings suggest that spatial receptive fields may become narrower during task performance also in the human auditory cortex and that this effect can be induced crossmodally.

The enhancements in spatial selectivity found here were not specific to the direction of visual orientation. Further, in the previous cat study these enhancements occurred also during a listening task in which spatial location was not relevant (Lee and Middlebrooks, 2011). This suggests that the changes in spatial coding in auditory cortex might not be exclusively due to attention to spatial locations. Instead, they may be an outcome of a higher level of arousal during a demanding task than during idle listening. However, general arousal does not seem to have an effect on the sensitivity to other sound features than spatial location. Previous EEG and MEG studies have recorded sensitivity to changes in the frequency patterns of sounds during the performance of a visual task (for instance, Sculthorpe et al., 2008; Chait et al., 2011). These studies have found no enhancements in neural sensitivity when the subject is engaged in a demanding visual task compared to idle listening or an easier task. This suggests that if general arousal is capable of enhancing auditory sensitivity, the effect is specific to spatial selectivity instead of being a general enhancement in all auditory processing. An enhancement in auditory spatial processing may have great survival value. The auditory system provides information on objects and events in locations outside the visual field and in distant locations. Based on this information other sensory modalities and motor functions can be directed. Therefore, in demanding situations causing an elevated level of arousal, enhanced processing of auditory spatial information may be beneficial.

Here, the enhancement in spatial selectivity did not depend on the direction of visual orientation. Also to our knowledge, no previous reports exist on local enhancements in auditory spatial selectivity for attended locations in behavioral or neural experiments. Therefore, one may consider the possibility that the auditory cortex is not capable of providing such enhancements. In the auditory cortex, sound source location is represented by a population rate code that consists of two opponent populations, one tuned to the left and the other to the right hemifield (Stecker et al., 2005; Werner-Reiss and Groh, 2008; Salminen et al., 2009, 2012). In this code, there are no single neurons dedicated to a restricted range of locations. Therefore, there are no single neurons whose selectivity could exclusively be enhanced to produce a local improvement in sensitivity. However, recent animal studies have described an alternative mechanisms through which the population rate code might be able to target better selectivity to specific spatial locations. This has been described in subcortical auditory neurons as an adaptation mechanism to stimulation statistics (Dahmen et al., 2010; Maier et al., 2012). In the wide spatial tuning curves, the best discrimination power between sound source locations is provided by the steepest parts of the slopes. These coincide normally with locations directly in front (Stecker et al., 2005). In the subcortical neurons described in these previous studies, the slope of the tuning curve tended to approach the location from which the majority of sounds originated (Dahmen et al., 2010; Maier et al., 2012). Thereby, these neurons shift their best discrimination ability toward the direction in which most of the sound sources reside. Hypothetically, orienting in space could have a similar effect, that is, the slope of the spatial tuning curves could migrate toward the direction of spatial attention. It remains, however, unclear whether such effects can take place in the human brain or in response to spatial selective attention.

One possible reason why location-specific modulation in auditory spatial tuning has not been found so far is that the enhancement may occur only during very specific task conditions. The present and previous studies (Ahveninen et al., 2006; Lee and Middlebrooks, 2011) have utilized tasks that do not require the subject to discriminate between sound source locations in one specific direction. Either spatial attention has been distributed or the task has involved other stimulus properties than spatial location in the attended direction. In such tasks, a local improvement in neural selectivity to location is not directly beneficial to the performance of the task. Since such a modulation does not improve task performance it may not take place. Therefore, in order to seek for neural correlates of spatial selective attention in the future, it will be important to craft the behavioral task so that the neural modulation searched for is directly beneficial for task performance.

Here, no gain effect was found related to the direction of visual orientation: responses to the sound in the left were of the same amplitude when the subjects oriented visually to the left and to the right. Previous EEG studies have found larger negativity in the ERPs for sounds originating from visually attended locations than from unattended ones (Eimer and Schröger, 1998; Teder-Sälejärvi et al., 1999). These increases have, however, occurred at longer latencies (150–400 ms) than the N1m response (peaking at 150 ms or earlier) under investigation in the present study. It may well be that the gain effect arises from some other mechanism than the feature-selective neurons whose activity the stimulus-specific adaptation presumably reflects. Further, it is hard to imagine how the spatially selective neurons in the auditory cortex with their wide receptive fields (approximately 180°, Stecker et al., 2005) could produce gain effects that are restricted to narrow ranges of locations (less than 20°, Teder-Sälejärvi and Hillyard, 1998). This suggests that the gain effect, at least for sound source location, may be due to some other neural mechanism.

In conclusion, the present results show that the representation of auditory space in the human cortex can be modulated not only by auditory but also by visual tasks. Together with previous studies, our findings suggest that enhancements in auditory spatial selectivity can occur under diverse conditions, even when sounds or spatial locations are not relevant to the task. This suggests that the population rate code of auditory space is sensitive to the state of the subject perhaps in order to provide improved location information of all potentially relevant events in the environment under demanding conditions.

### Conflict of interest statement

The authors declare that the research was conducted in the absence of any commercial or financial relationships that could be construed as a potential conflict of interest.

## References

[B1] Ahveninen J. Jääskeläinen I. P. Raij T. Bonmassar G. Devore S. Hämäläinen M. (2006). Task-modulated “what” and “where” pathways in human auditory cortex. Proc. Natl. Acad. Sci. U.S.A. 103, 14608–14613 10.1073/pnas.0510480103 16983092 PMC1600007

[B2] Briley P. M. Kitterick P. T. Summerfield A. Q. (2013). Evidence for opponent process analysis of sound source location in humans. J. Assoc. Res. Otolaryngol. 14, 83–101 10.1007/s10162-012-0356-x 23090057 PMC3540274

[B3] Butler R. A. (1972). The influence of spatial separation of sound sources on the auditory evoked response. Neuropsychologia 10, 219–225 10.1016/0028-3932(72)90063-2 5055228

[B4] Chait M. Ruff C. C. Griffiths T. D. McAlpine D. (2011). Cortical responses to changes in acoustic regularity are differentially modulated by attentional load. Neuroimage 59, 1932–1941 10.1016/j.neuroimage.2011.09.006 21945789 PMC3271381

[B5] Dahmen J. C. Keating P. Nodal F. R. Schulz A. L. King A. J. (2010). Adaptation to stimulus statistics in the perception and neural representation of auditory space. Neuron 66, 937–948 10.1016/j.neuron.2010.05.018 20620878 PMC2938477

[B6] Eimer M. Schröger E. (1998). ERP effects of intermodal attention and cross-modal links in spatial attention. Psychophysiology 35, 313–327 9564751 10.1017/s004857729897086x

[B7] Grothe B. Pecka M. McAlpine D. (2010). Mechanisms of sound localization in mammals. Physiol. Rev. 90, 983–1012 10.1152/physrev.00026.2009 20664077

[B8] Hillyard S. A. Hink R. F. Schwent V. L. Picton T. W. (1973). Electrical signs of selective attention in the human brain. Science 182, 177–180 10.1126/science.182.4108.177 4730062

[B9] Lee C.-C. Middlebrooks J. C. (2011). Auditory cortex spatial sensitivity sharpens during task performance. Nat. Neurosci. 14, 108–114 10.1038/nn.2713 21151120 PMC3076022

[B10] Magezi D. A. Krumbholz K. (2010). Evidence for opponent-channel coding of interaural time differences in human auditory cortex. J. Neurophysiol. 104, 1997–2007 10.1152/jn.00424.2009 20702739 PMC2957465

[B11] Maier J. K. Hehrmann P. Harper N. S. Klump G. M. Pressnitzer D. McAlpine D. (2012). Adaptive coding is constrained to midline locations in a spatial listening task. J. Neurophysiol.108, 1856–1868 10.1152/jn.00652.2011 22773777 PMC4422344

[B12] Middlebrooks J. C. Green D. M. (1991). Sound localization by human listeners. Annu. Rev. Psychol. 42, 135–159 10.1146/annurev.ps.42.020191.001031 2018391

[B13] Palomäki K. J. Tiitinen H. Mäkinen V. May P. J. Alku P. (2005). Spatial processing in human auditory cortex: the effects of 3D, ITD, and ILD stimulation techniques. Brain Res. Cogn. Brain Res. 24, 364–379 10.1016/j.cogbrainres.2005.02.013 16099350

[B14] Salminen N. H. May P. J. Alku P. Tiitinen H. (2009). A population rate code of auditory space in the human cortex. PLoS ONE 4:e7600 10.1371/journal.pone.0007600 19855836 PMC2762079

[B15] Salminen N. H. Tiitinen H. May P. J. (2012). Auditory spatial processing in the human cortex. Neuroscientist 18, 602–612 10.1177/1073858411434209 22492193

[B16] Sculthorpe L. D. Collin C. A. Campbell K. B. (2008). The influence of strongly focused visual attention on the detection of change in an auditory pattern. Brain Res. 1234, 78–86 10.1016/j.brainres.2008.07.031 18674520

[B17] Stecker G. C. Harrington I. A. Middlebrooks J. C. (2005). Location coding by opponent neural populations in the auditory cortex. PLoS Biol. 3:e78 10.1371/journal.pbio.0030078 15736980 PMC1044834

[B18] Teder-Sälejärvi W. A. Hillyard S. A. (1998). The gradient of spatial auditory attention in free field: an event-related potential study. Percept. Psychophys. 60, 1228–1242 9821784 10.3758/bf03206172

[B19] Teder-Sälejärvi W. A. Muente T. F. Sperlich F.-J. Hillyard S. A. (1999). Intra-modal and cross-modal spatial attention to auditory and visual stimuli. An event-related brain potential study. Brain Res. Cogn. Brain Res. 8, 327–343 10.1016/S0926-6410(99)00037-3 10556609

[B20] Werner-Reiss U. Groh J. M. (2008). A rate code for sound azimuth in monkey auditory cortex: Implications for human neuroimaging studies. J. Neurosci. 28, 3747–3758 10.1523/JNEUROSCI.5044-07.2008 18385333 PMC3654685

[B21] Zatorre R. J. Penhune V. B. (2001). Spatial localization after excision of human auditory cortex. J. Neurosci. 21, 6321–6328 11487655 10.1523/JNEUROSCI.21-16-06321.2001 PMC6763137

